# Polymer- and Lipid-Based Nanostructures for Wound Healing with Barrier-Resolved Design

**DOI:** 10.3390/pharmaceutics17111501

**Published:** 2025-11-20

**Authors:** Eunsoo Cho, Soyeon Yun, Subin Lee, Minse Kim, Jaewon Choi, Sun Eun Choi, Kwang Suk Lim, Suk-Jin Ha, Jang-Hyuk Yun, Hyun-Ouk Kim

**Affiliations:** 1 Division of Chemical Engineering and Bioengineering, College of Art, Culture and Engineering, Kangwon National University, Chuncheon-si 24341, Gangwon-do, Republic of Korea; joeunsoo@kangwon.ac.kr (E.C.); isubin0903@kangwon.ac.kr (S.L.); minse0415@kangwon.ac.kr (M.K.); choijw8839@kangwon.ac.kr (J.C.); kslim@kangwon.ac.kr (K.S.L.);; 2 Department of Molecular Bioscience, College of Biomedical Sciences, Kangwon National University, Chuncheon-si 24341, Gangwon-do, Republic of Korea; king030619@kangwon.ac.kr; 3 Department of Smart Health Science and Technology, Kangwon National University, Chuncheon-si 24341, Gangwon-do, Republic of Korea; 4 Institute of Industrial Technology, Kangwon National University, Chuncheon-si 24341, Gangwon-do, Republic of Korea; 5 Department of Forest Biomaterials Engineering, Kangwon National University, Chuncheon-si 24341, Gangwon-do, Republic of Korea; oregonin@kangwon.ac.kr; 6 Dr.Oregonin Inc., #802 Bodeum Hall, Kangwondaehakgil 1, Chuncheon-si 24341, Gangwon-do, Republic of Korea; 7 Institute of Fermentation of Brewing, Kangwon National University, Chuncheon-si 24341, Gangwon-do, Republic of Korea; 8 College of Veterinary Medicine and Institute of Veterinary Science, Kangwon National University, Chuncheon-si 24341, Gangwon-do, Republic of Korea

**Keywords:** wound healing, lipid-based nanoparticles, polymer-based nanostructures, polymer–lipid hybrid vesicles, smart wound dressings

## Abstract

Chronic and hard-to-heal wounds remain burdensome because microbial contamination, dysregulated inflammation, and fragile tissue regeneration slow closure, while passive dressings often injure new tissue during removal. This review synthesizes polymer- and lipid-based nanostructures through a barrier-resolved lens that links composition, architecture, and processing to performance in protease- and salt-rich exudate across topical and transdermal routes. Quantitative trends include effective diameters of approximately 50–300 nm, practical constraints of sterile filtration at 0.2 μm, and therapeutic windows that prioritize contamination control on the first day, support proliferation around day three, and sustain remodeling beyond one week. Mechanistic evidence indicates that interfacial charge and the protein corona govern residence and uptake, lipid bilayers enable dual loading, degradable polymer matrices provide depot-like behavior, and hybrid constructs temper the early burst while improving storage stability.

## 1. Introduction

**Scheme 1:**
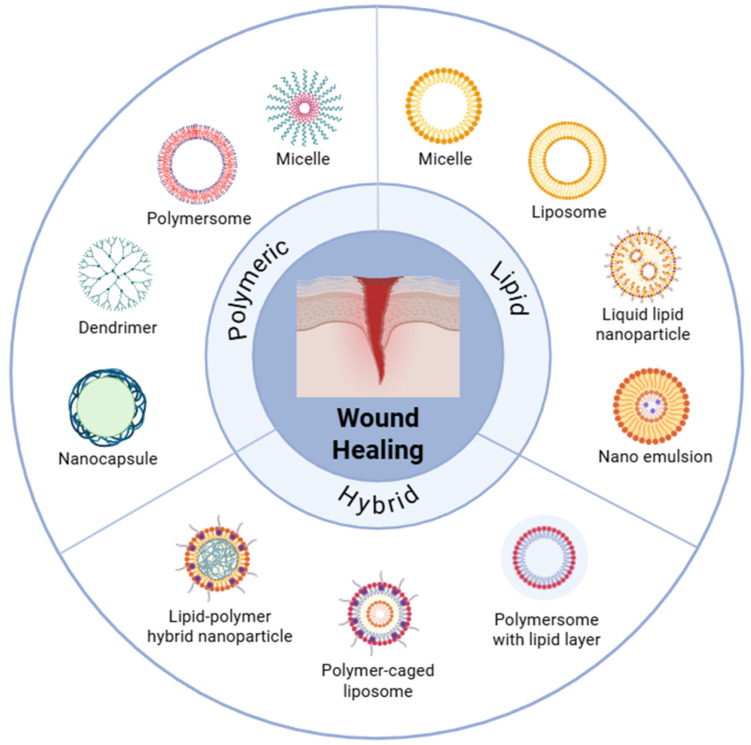
Nanostructures for wound healing can be categorized into three groups: polymeric, lipid-based, and hybrid. Some examples of representative systems are polymersome, micelle, dendrimer, nanocapsule, liposome, solid lipid nanoparticle, nanostructured lipid carrier, and polymer–lipid hybrid vesicle. The plan stresses how each structure is built to help the skin heal, control inflammation, and make it easier for drugs to enter and stay in.

Chronic wounds that are hard to heal put a lot of stress on the healthcare system and the economy because they cause a lot of problems with microbial load, inflammation that is not working correctly, and tissue regeneration that is not working correctly. Conventional dressings only provide passive coverage and can hurt fragile granulation when they are taken off [1]. Polymer- and lipid-based nanostructures have arisen as promising wound care carriers capable of actively modulating local antimicrobial delivery, moisture retention, and regenerative signaling within the wound bed. However, many formulations are mechanically fragile, do not stay hydrated for long, and break down when they come into contact with enzyme- and salt-rich exudates. This makes them less effective in clinical settings over time [2]. Scheme 1 organizes the principal carrier families into polymeric, lipid-based, and hybrid classes to frame how architectural and interfacial design choices align with the needs of the wound bed.

**Figure 1:**
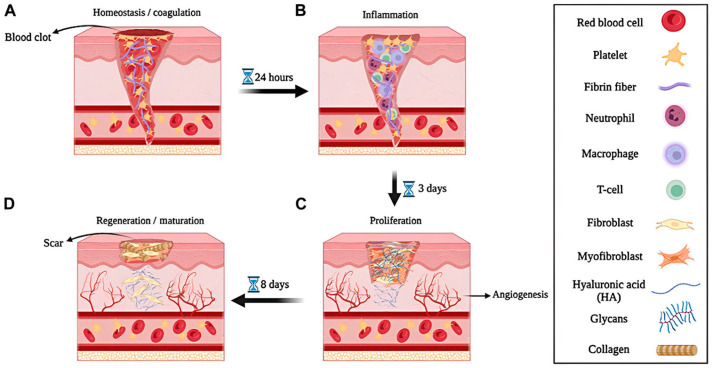
Multiscale illustration and experimental validation of the wound healing process. Schematic representation of four consecutive wound healing phases: (**A**) Hemostasis and coagulation, (**B**) inflammation, (**C**) proliferation, and (**D**) regeneration/maturation, with major cellular and extracellular components indicated. Reprintied with permission from Ref. [5]. 2024, Biotechnology.

Many studies report a single hydrodynamic size and a single surface charge measured in water, which obscures behavior in albumin- and mucin-rich media and complicates dose planning and cross-platform comparisons [3]. In addition, animal models that close by contraction underrepresent ischemia, neuropathy, and polymicrobial biofilm [4]. Figure 1 situates delivery within the staged biology of hemostasis, inflammation, proliferation, and maturation, underscoring the need for time-matched exposure that suppresses early contamination while supporting re-epithelialization, angiogenesis, and matrix organization [5].

This review advances a barrier-resolved framework grounded in the premise that particle size distributions, interfacial charge in wound-like media, and internal architecture can be tuned to synchronize early antimicrobial availability with later pro-regenerative signaling, while preserving comfort on removal [6]. We outline design principles for polymer matrices, lipid vesicles, and polymer–lipid hybrids, and we propose a validation path that begins in human exudate surrogates, proceeds through ex vivo human skin and porcine full-thickness models, and links analytical attributes to outcomes including time to closure, reduction in biofilm burden, re-epithelialization, angiogenesis, collagen organization, and pain during dressing changes [7]. To facilitate reliable clinical translation, this approach emphasizes the transparent reporting of number-based size and shape distributions, surface charge measured in protein- and salt-rich media, and chemical integrity indicators such as oxidation markers and residual solvent content, alongside post-sterilization stability tests to ensure reproducibility and regulatory compliance [8].

## 2. Determinants and Barrier Interactions of Polymer- and Lipid-Based Nanostructures in Wound

### 2.1. Physicochemical Determinants

Polymer-, lipid-, and hybrid-based nanostructures are the three main types of trans-porters that help wounds heal (Scheme 1). Nanoparticles made of polymers, like PLGA, PEG, and chitosan, make matrices that can be solid or gel-like. You can change these matrices so that they break down at different rates for mechanical support, controlling moisture, and releasing their contents over time [9,10]. Lipid-based nanoparticles, such as liposomes, solid lipid nanoparticles, and nanostructured lipid carriers, resemble biological membranes. They are very biocompatible, can make contact with wet wounds in a way that is not too rough, and can hold two drugs at the same time in both aqueous and lipid domains [11,12]. Polymer–lipid hybrids combine the structural strength of polymers with the biomimetic interfaces of lipids to give you balanced stability, permeability, and explosive control [13,14]. These architectures establish the basis for understanding how physical–chemical determinants such as size, charge, and internal structure regulate performance in protease and salt-rich wound environments.

Polymer and lipid-based nanostructures for wound healing exhibit size, charge, and structure, physicochemical features that control hydration, tissue penetration, and stability in proteases and salt-rich exudates. The 50–300 nm size generally favors follicle entry and interstitial transport. A near-neutral surface reduces irritation, and a cationic surface improves adhesion but increases the risk of opsonization. Lipid vesicles guarantee contact with moist wounds and enable dual drug loading. Biodegradable polymers provide mechanical strength and continuous release, and the hybrid system combines the polymer core and lipid shell to bind for explosion control and ligand attachment, but changes in batch and sterile stability. Recent studies show that this size range can change depending on the situation. Hybrid and inorganic polymer systems can be effectively maintained outside of the usual 50–300 nm range, depending on the carrier composition and the microenvironment of the wound. For example, curcumin–cyclodextrin hybrid nanoparticles (about 150 nm) help the skin cells grow back and speed up the healing process [15]. PEG-coated magnesium hydroxide nanoparticles also helped cells absorb more of the drug and speed up the healing of wounds [16]. These results show that particle size should be adjusted along with surface charge, corona behavior, and payload chemistry, rather than being treated as a constant.

Table 1 shows that nanoparticles made of polymers, lipids, and hybrids have different physical and chemical properties that affect how well they heal wounds. Polymer-immersed carriers can be made larger or smaller by changing the polymer composition and processing. This ensures the strong adhesion to mucus and long-lasting release, but they can only hold a limited amount of hydrophilic material and may produce byproducts that break down in acid. Lipid-based systems are biocompatible and can mimic membranes and make contact with wet wound surfaces, but they can also change phases and become unstable in serum.

### 2.2. Biocompatibility and Tissue Adhesion Profiles

**Figure 2:**
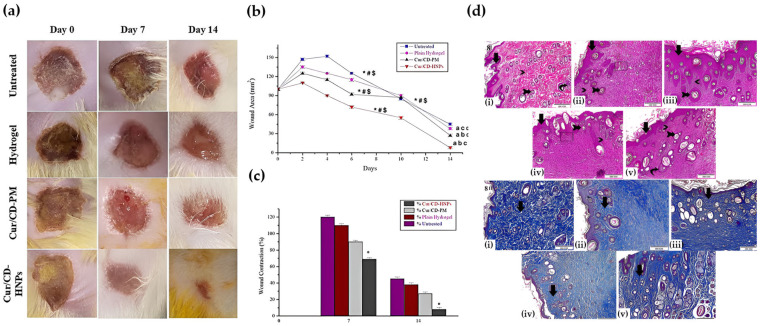
In vivo and histological evaluation of Cur/CD-HNPs demonstrating biocompatibility and tissue adhesion in burn wound healing. (**a**) Representative wound photographs taken on days 0, 7, and 14 for the untreated, hydrogel, Cur/CD-PM, and Cur/CD-HNP groups. (**b**) Quantitative analysis of wound area reduction over 14 days. Statistical symbols indicate significant differences at each time point: * *p* < 0.05 vs. untreated; # *p* < 0.05 vs. Cur/CD-PM; $ *p* < 0.05 vs. hydrogel. (**c**) Wound contraction (%) on days 7 and 14. (**d**) Histopathological evaluation of skin tissues: (**i**–**v**) H&E staining showing re-epithelialization, reduced edema, and regeneration of dermal and adnexal structures—including newly formed epidermal layers, restored hair follicles, and diminished inflammatory cell infiltration—highlighted by black arrows; (**i**–**v**) Masson’s Trichrome staining indicating organized collagen deposition and restored connective tissue architecture in the Cur/CD-HNP treatment group. Reprinted with permission from Ref. [15]. 2025, BMC Biotechnology.

Lipid carriers that include liposomes, solid lipid nanoparticles, and nanostructured lipid carriers often display high tolerability on skin because their membrane-mimetic composition aligns with native barriers and supports interaction with keratinocytes and macrophages while limiting disruption of the stratum corneum, although stability can decline in protease- and salt-rich exudate, and repeated application can amplify complement signals and irritation. PLGA, PEG, and chitosan are examples of degradable polymer matrices that can be adjusted to lower inflammation and balance moisture by controlling the flow of water vapor. Chitosan is a strong adhesive that keeps the matrix on exudative wounds longer, but it also makes it harder for the matrix to penetrate dense extracellular matrices and makes it harder to remove without causing trauma [18,19]. Polymer–lipid hybrids have a soft lipid interface that helps cells connect with them and a supportive polymer core that keeps them from leaking. But because the architecture is more complicated, it is harder to keep batches consistent, sterilize them, and store them safely. This means that early process controls are necessary [20,21,22]. Many studies continue to employ simplified media and short exposures that fail to accurately depict cumulative irritation, cytokine release, complement activation, and the interference caused by mucins and reactive oxygen species in wound fluid. Consequently, decision-ready evidence must encompass hemolysis, complement, cytotoxicity, irritation, and transport assays conducted in wound-like media, alongside barrier-resolved outcomes that accurately reflect biofilm penetration, re-epithelialization kinetics, macrophage polarization, and scar quality under equivalent dose and exposure conditions [23,24,25]. The in vivo performance of Cur/CD-HNPs corroborates the aforementioned biocompatibility and adhesion properties (Figure 2). In a burn wound model, Cur/CD-HNPs significantly expedited the healing process in comparison to the untreated, hydrogel, and Cur/CD-PM groups. The series of pictures in Figure 2a clearly shows that the Cur/CD-HNPs group closed their wounds and re-epithelialized quickly by day 14. Quantitative analyses (Figure 2b,c) demonstrated a reduction in the wound area and an increase in the contraction ratios, thereby confirming the acceleration of the healing process. Histopathological evaluation (Figure 2d) further confirmed the tissue’s compatibility and regenerative capacity. The H&E staining showed that the epidermal layers were still there and that the swelling had gone down. Masson’s Trichrome staining, on the other hand, showed that the dermis’s structure was restored and that the collagen fibers were dense and organized. These results show that hybrid nanoparticles are not harmful to cells, reduce irritation, and encourage tissue adhesion and regeneration at the same time. This shows that they are good at transporting medicine to wounds.

### 2.3. Degradation and Controlled-Release Profiles

Diffusion through lipid assemblies and hydrolytic erosion of polymer matrices determine when and where therapeutic payloads are accessible within the wound bed. The lactic-to-glycolic ratio, molecular weight, crystallinity, hydration, pH, and temperature of the exudate all affect the ester cleavage of PLGA and related copolymers. But if buffering is not used, acidic byproducts can lower the pH locally and irritate tissue around wounds [26]. The permeability of lipid carriers varies according to the presence of ionizable headgroups, cholesterol content, and acyl chain order. Their integrity can deteriorate in pro-tease- and salt-rich fluid or under dehydration and shear during dressing changes [27]. Hybrid polymer–lipid constructs often temper early burst through a bilayer shell while a polymer core functions as a reservoir. But interfacial defects, drying steps, and sterilization can cause batch variability and leaks that cannot be predicted [21]. The release profile must align with the temporal requirements necessary for healing. During the initial control of contamination, it is important to have quick access to antimicrobials. During granulation and remodeling, on the other hand, it is important to have ongoing signals that promote anti-inflammation or regeneration. But if exposed to the sun for too long, fibroblast migration can be slowed down, and reepithelialization can take longer [28]. Numerous studies continue to define release in uncomplicated buffers under static conditions. These conditions do not include enzymes, mucins, albumin, or reactive oxygen species, and they do not take into account cyclic hydration or shear. As a result, the apparent stability can be inflated, obscuring dosing intervals and dressing replacement plans. Therefore, evaluation should adopt exudate-mimicking media with proteolytic and oxidative challenges, report burst fraction and half-life at a matched dose and surface area-to-volume ratio, include testing under hydration–dehydration cycles, and link in vitro kinetics to barrier-resolved outcomes such as biofilm reduction, macrophage polarization, collagen organization, and rate of re-epithelialization [29,30].

### 2.4. Dimensional and Interfacial Parameters

Size, surface charge, and morphology govern how polymer- and lipid-based nanostructures distribute within wound tissue, interact with exudate, and deliver cargo to keratinocytes, fibroblasts, and macrophages, with sub-100 nm fractions more capable of traversing follicular routes and biofilm channels, and larger particles favoring local depot behavior with reduced spread [31,32]. Near-neutral or slightly negative interfaces tend to limit nonspecific protein adsorption, complement binding, and irritation, while the controlled inclusion of ionizable lipids or cationic polymers can improve endosomal release through pH-responsive interactions at the expense of stronger mucus trapping and opsonization [33,34]. Spherical and smooth morphologies generally support storage stability and predictable transport, whereas anisotropic or rough surfaces increase contact with irregular wound beds and can heighten phagocytic clearance [35]. These design levers are often constrained because many studies report only a single hydrodynamic diameter in water and a single zeta potential without ionic strength, temperature, or exudate composition, with sparse disclosure of number-based distributions and electron microscopy descriptors, and with comparisons performed at unequal nucleic acid dose and N-to-P ratio, which leaves platform selection uncertain for outcomes such as biofilm penetration, re-epithelialization, and scar quality [36,37]. Decision-ready characterization should include full size and shape distributions with polydispersity, zeta potential measured in exudate-mimicking media that contain albumin, mucins, salts, and reactive oxygen species, explicit buffer and temperature reporting, and performance under cyclic hydration and shear that reflects dressing changes while holding nucleic acid dose and N-to-P ratio constant across formulations [38,39].

## 3. Fabrication and Surface Engineering of Polymer- and Lipid-Based Nanostructures

### 3.1. Polymer Nanoparticle Fabrication Techniques

Polymer nanoparticles (PNPs) for wound healing are produced from preformed polymers or through the in situ polymerization of monomers, with selection guided by the target size profiles, payload compatibility, residual and endotoxin limits, sterilization plan, and intended scale [40,41]. Preformed routes include solvent evaporation, nanoprecipitation, single or double emulsion, dialysis, salting out, and supercritical methods such as RESS (rapid expansion of supercritical solutions) and RESOLV (rapid expansion of supercritical solution into liquid solvent) [42]. These methods enable broad material choice and tunable loading but require the stringent removal of solvent and surfactant to avoid cytotoxicity in protease- and salt-rich exudate, as well as requiring careful control of polydispersity and batch variability [33]. Emulsion, mini or microemulsion, surfactant-free emulsion, interfacial polymerization, and controlled radical polymerization using RAFT (reversible addition fragmentation chain transfer) and ATRP (atom transfer radical polymerization) are all examples of in situ methods [43]. These methods make sure that the chain structure and depot are correct, but they also come with risks from leftover monomers and initiators; they need to follow strict purification and release standards [44]. PLGA and PEG allow for an adjustable release design. On the other hand, microfluidic mixing with continuous manufacturing may help with size dispersion, but it will slow down throughput [45]. Many studies still test particles in deionized water under static conditions that omit enzymes, albumin, mucins, and reactive oxygen species; therefore, method reports should include number-based size and shape distributions, zeta potential in exudate-like media, residual and endotoxin limits, and post-sterilization stability linked to re-epithelialization, biofilm control, and scar quality [46,47].

#### 3.1.1. Emulsion–Solvent Evaporation

PNPs for wound healing are frequently prepared by emulsion–solvent evaporation, in which PLGA, PLA, or PCL with payload is dissolved in a volatile organic phase such as EA or DCM, dispersed into an aqueous phase containing PVA by high shear or ultrasound, and then solidified by solvent removal through stirring, mild heating, or vacuum [47,48]. O/W emulsions suit hydrophobic cargos, while W/O/W double emulsions enable proteins and NAs [49]. The solvent choice, polymer and PVA concentration, energy input, and temperature govern size distribution, porosity, and EE, with implications for burst and depot behavior in exudate [50]. The strengths include broad material compatibility and simple equipment. The limitations include residual solvent and PVA, batch variability, shear-induced protein loss at interfaces, and modest loading of highly water-soluble agents [51]. Numerous studies purify and characterize particles in deionized water, which exaggerates perceived stability and minimizes irritation risk in environments rich in proteases and salts [52]. Development must validate mass balance and residuals in accordance with wound-specific thresholds, regulate endotoxin levels, ensure stability post-terminal sterilization, and be evaluated in exudate-like media containing enzymes, mucins, albumin, and reactive oxygen species (ROS) to correlate process outputs with outcomes including biofilm management, re-epithelialization, and scar quality [53,54].

#### 3.1.2. Nanoprecipitation

Nanoprecipitation produces PNPs by injecting an organic solution of PLA, PLGA, or PCL with a hydrophobic payload into an aqueous non-solvent under rapid mixing, where solvent–water diffusion drives polymer desolvation, nucleation, and development into nanospheres, while the inclusion of an oil phase yields nanocapsules. Water-miscible solvents such as ACN or EA and stabilizers such as TPGS or PVA shape interfacial tension and suppress aggregation. A number of controllable factors have a clear effect on the final particle size and polydispersity. For example, lower concentrations lead to more nucleation events and smaller, more uniform particles, while higher concentrations of polymers lead to more local supersaturation during growth-dominated regimes, which results in a larger and broader PDI [55]. Supersaturation kinetics are significantly influenced by the solvent-to-water ratio; a higher aqueous fraction promotes growth or aggregation and slows desolvation, while a higher solvent content speeds up the diffusion and nucleation and produces smaller particles [56]. Another important factor is the injection or flow rate: slow injection promotes diffusion-limited growth and wider distributions, whereas rapid injection produces steep concentration gradients and a large number of nuclei, which reduce particle size [57]. Elevated temperature increases diffusion and decreases viscosity, generally favoring smaller particles, though excessive heating can destabilize emulsions or induce secondary aggregation [58]. Likewise, high-energy mixing or shear—through turbulent stirring or microfluidic confinement—shrinks diffusion boundary layers, producing smaller and more uniform particles, whereas low-energy mixing often yields multi-modal size distributions [59].

**Figure 3:**
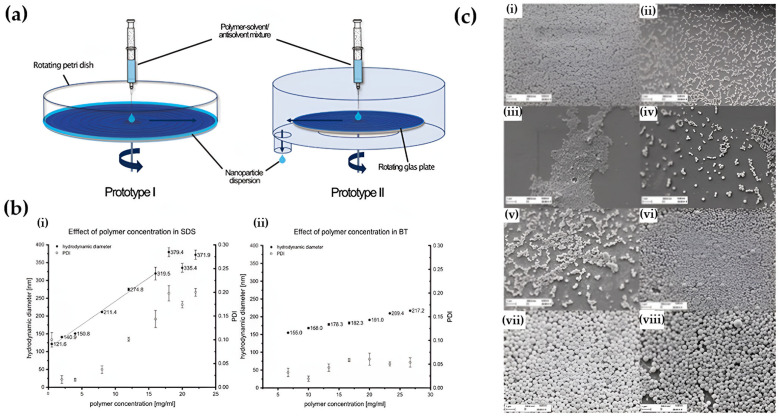
Mechanism and parameter effects in nanoprecipitation of PLGA nanoparticles. (**a**) Schematic of evaporation-triggered nanoprecipitation illustrating polymer–solvent/antisolvent mixing and nucleation on a rotating substrate. (**b**) Influence of polymer concentration and stabilizer type on hydrodynamic diameter and polydispersity index (DLS), demonstrating the shift from nucleation-dominated to growth-dominated regimes. (**c**) Representative SEM images showing how polymer molecular weight, lactic/glycolic ratio, and solvent/antisolvent combinations modulate nanoparticle morphology and size distribution: (**i**) Low–molecular-weight PLGA (7–17 kDa, 50:50) in ACE/H_2_O producing larger, irregular particles. (**ii**) Medium–molecular-weight PLGA (24–38 kDa, 50:50) forming smaller and more uniform nanospheres. (**iii**) High–molecular-weight PLGA (38–54 kDa, 50:50) yielding highly uniform and smaller particles due to reduced chain mobility. (**iv**) Increased LA:GA ratio (65:35) resulting in larger and less monodisperse particles. (**v**) Higher LA content (75:25) further enlarging particle size and broadening PDI. (**vi**) ACE/EtOH mixtures producing larger, less uniform nanoparticles due to slower desolvation kinetics. (**vii**) ACN/H_2_O conditions yielding the largest particles owing to rapid solvent diffusion and reduced nucleation density. (**viii**) Optimized ACE/H_2_O conditions producing the smallest and most spherical particles, reflecting efficient nucleation and narrow size distributions. Reprinted with permission from Ref. [61]. 2025, Drug Delivery Science and Technology.

Because nanoprecipitation outcomes reflect the coupled kinetics of nucleation and growth, parameter sweeps (polymer concentration, solvent–water ratio, flow rate, temperature, and mixing intensity) should be explicitly linked to particle size, polydispersity, and encapsulation efficiency. Protease- and salt-rich exudate can be irritated by residual solvent and surfactant, and stability in DI water under static conditions is rarely a reliable indicator of performance under cyclic hydration and shear. Therefore, the zeta potential and release profiles in exudate-like media, mass balance for loading and recovery, and post-sterilization stability should all be included in decision-ready reports [60]. A representative process and parameter effects are illustrated in Figure 3. Evaporation-triggered nanoprecipitation involves injecting a polymer–solvent/antisolvent mixture onto a rotating substrate, where rapid solvent diffusion induces polymer desolvation and nucleation (Figure 3a). Dynamic light scattering (Figure 3b) shows that the type of stabilizer and the concentration of the polymer have a big effect on the hydrodynamic diameter and the polydispersity index. As the concentration of polymers rises, regimes dominated by growth emerge, resulting in a higher PDI, which aligns with the previously discussed supersaturation kinetics. Scanning electron microscopy shows different shapes of nanoparticles and how they stay the same under different polymer and solvent conditions (Figure 3c). This confirms that the best compositions make spherical and monodisperse PLGA nanoparticles [61].

#### 3.1.3. Electrospinning Nanofiber Fabrication

Electrospinning makes meshes that help wounds heal by pulling a charged polymer jet from a syringe needle to a grounded collector while the solvent evaporates. This makes fibers with a large surface area that can be changed in diameter, alignment, and porosity [62]. When making solutions of PCL, PLA, PLGA, PEO, or mixtures that contain antimicrobials or growth factors, the voltage, flow rate, and tip-to-collector distance must be just right. The temperature and humidity also affect the whipping, crystallinity, and web integrity [63]. Coaxial, triaxial, melt, multi-jet, and needleless formats allow for core–shell loading, solvent-free operation, and higher throughput. Rotating or patterned collectors set alignments that can help keratinocyte and fibroblast migration [64]. The benefits include conformal contact with irregular wounds and continuous delivery from a mechanically compliant depot [65]. Constraints remain, including jet instability, limited industrial yield, and residual solvent that can irritate protease- and salt-rich exudate, while early hydration can trigger bursting when crystallinity or crosslinking is inadequate [66]. Many reports present mean fiber diameter from dry images and elution in DI media, which overstates mechanical and release stability. Decision-ready method sections should disclose diameter distributions and pore fraction, surface charge, drug localization, residuals and endotoxins, wet tensile behavior, post-sterilization stability, and burst and antimicrobial performance in exudate-like media under cyclic hydration [67,68].

### 3.2. Lipid Nanostructure Engineering

**Figure 4:**
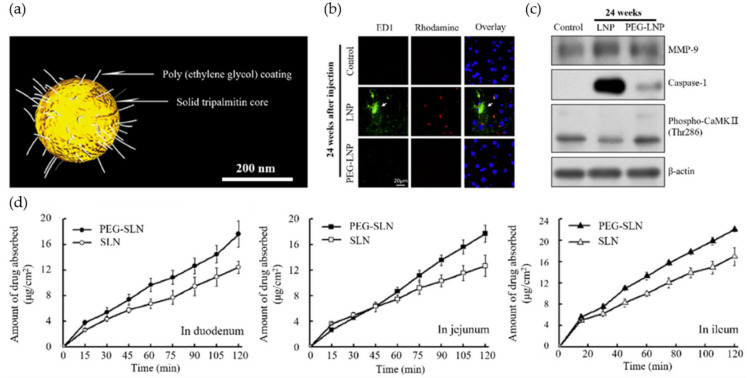
LNP functionality modulated by polymer-based surface modification. (**a**) Schematic illustration of PEG-stabilized LNPs composed of a solid tripalmitin core and a PEG coating. (**b**) Immunofluorescence images showing mitigation of neurovascular damage following PEG-LNP administration. FD1 (green) visualizes fluorescein–dextran leakage across the vascular barrier, Rhodamine (red) labels vascular structures, and DAPI (blue) stains nuclei. PEGylation reduces FD1 extravasation, indicating improved neurovascular integrity. (**c**) Immunoblot analysis of mouse brain tissue 24 h after injection, probed for MMP-9 (blood–brain barrier integrity), caspase-1 (inflammation), and phospho-CaMKII (synaptic signaling), with β-actin as a loading control. (**d**) Absorptive characteristics of SLN and PEG-SLN in the everted rat gut sac system across the duodenum, jejunum, and ileum, showing enhanced and more consistent absorption for PEG-SLN. Reproduced with permission from Ref. [77]; Copyright 2023, Bioengineering.

LNP platforms that have SLN and NLC are made with HPH, emulsion, or microfluidics. The size, PDI, loading, and release depend on the composition and process [69,70]. When you mix solid and liquid lipids, you obtain an NLC with lattice defects that change the crystallinity and increase the capacity [71]. PEG makes colloids more stable, lowers the amount of protein that sticks to them, and lets ligands be added after insertion. But thicker coronas can make it harder for cells to touch each other and lower the amount of viscous exudate that cells take up [72]. LbL shells help control charge and protect enzymes, but they have some problems. For example, they do not let enough light through, they cannot dry out, and they cannot kill bacteria at the end [73]. Many researchers believe that sugars, peptides, or Ab fragments can actively target cells. But proteases, ROS, and biofilm matrices in wounds can break down ligands and hide epitopes, making them less useful [74]. Numerous studies demonstrate that DLS sizes in water and zeta function merely as fundamental buffers. They have not been tested with shear, albumin, or mucin yet. This makes it harder to compare reports and figure out how much to use [75]. PEG-LNPs help keep the interface stable, activate the immune system, and stop ions from crossing the epithelial barrier, as seen in Figure 3. This shows how important interfacial engineering is and how important it is to try using it in places that are like wounds and have changing levels of hydration and shear [76]. Figure 4 does not show us much about how PEG changes the way that lipid nanostructures work. Figure 4a shows a diagram with solid lipid cores and a PEG corona around them. This keeps the colloid from breaking down and keeps proteins from sticking to particles in biological fluids. PEGylation lowers immune activation and neuroinflammation, as shown by the drop in microglial activation and the restoration of neurovascular integrity (Figure 4b,c). The immunostaining of ED1-positive cells indicated that PEGylated LNPs inhibited microglial activation and inflammatory signaling. In contrast, immunoblotting showed that MMP-9, caspase-1, and phospho-CaMKII levels went down, which reduced neuroinflammation and kept synaptic signal transmission going. Furthermore, absorption studies employing an Everdead intestinal pocket model (Figure 4d) revealed that PEG-modified SLNs demonstrated enhanced and more consistent absorption in the duodenum, jejunum, and ileum compared to non-PEGylated SLNs. These results all show that modifying the interface of polymers makes lipid nanoparticles (LNPs) more stable, changes immune responses, and maintains epithelial transport, which shows how important it is to design lipid–polymer interfaces for use in wound healing [76].

#### 3.2.1. Liposome Fabrication Methods

**Figure 5:**
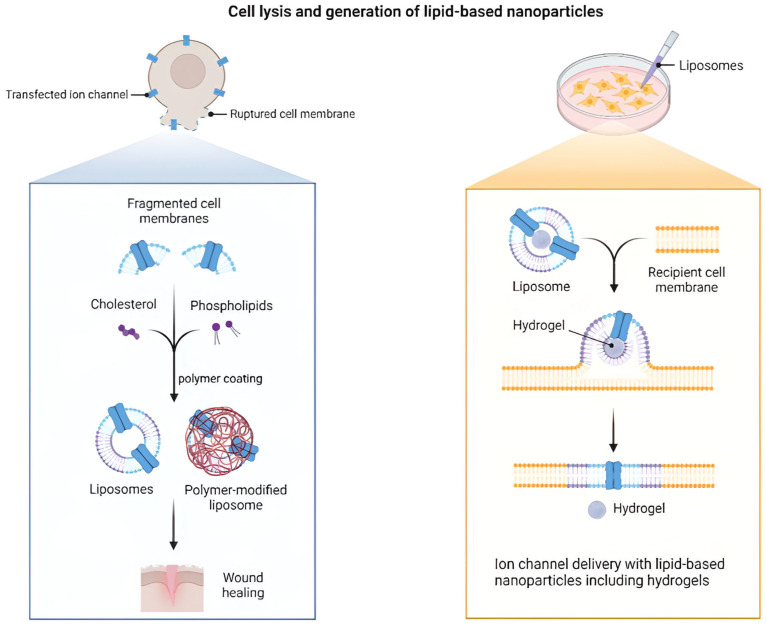
Cell lysis and LNP generation with hydrogel-assisted delivery. Panels show the following elements: TFH and EI production with size tuning by extrusion or HPH, optional PEG post-insertion membrane fragments providing cholesterol, and PL that assemble into liposomes or polymer-modified liposomes for wound healing, using hydrogel-assisted landing of a liposome on a recipient membrane with insertion of an ion channel [82]. Reproduced with permission from Ref. [82]; Copyright 2023, Functional Biomaterials.

TFH, EI, REV, DR, extrusion, HPH, microfluidic mixing, and SCF routes make liposomes for LNP platforms for wound care. The lipid mix, hydration conditions, and post-sizing define MLV or LUV populations, EE, and stability. TFH makes a dry film that is hydrated and shrunk by extrusion or HPH. EI injects a lipid solution into a buffer to quickly vesiculate it. REV makes W/O emulsions that collapse into large unilamellar vesicles with high aqueous loading. Finally, DR takes detergents out of mixed micelles to make homogeneous liposomes [78,79]. Adding PEG after insertion makes colloids more stable and less likely to stick to exudate that is high in protein. But thick coronas can make it harder for cells to touch each other and slow down uptake [80]. Microfluidics gives you a lot of control over mixing and size, but throughput and fouling limit the size. Previous studies often reported DLS size in water with insufficient details on oxidation markers, residual solvent, and batch variability, complicating the understanding of its functionality in enzyme- and salt-rich media [81]. Figure 4 illustrates the various pathways through which membrane-derived lipid sources, polymer-modified liposomes, and hydrogel-assisted delivery can reach recipient membranes. Development focused on wound environments should elucidate numerical size distributions, zeta potential in exudate-like media, lipid oxidation, and residual limits, alongside post-sterilization stability related to re-epithelialization and biofilm management [82]. Figure 5 shows how the mechanistic framework of membrane-derived lipid assembly and hydrogel-assisted delivery works. This figure shows that fragmented cell membranes with cholesterol and phospholipids can turn into lipid vesicles through thin-film hydration (TFH) or ethanol injection (EI). After that, the vesicles can be made smaller by extrusion or high-pressure homogenization (HPH). Adding PEG makes the colloidal stability even better and stops the protein-rich fluid that comes out of wounds from sticking together. Changing the polymer makes it stronger and easier to control how bioactive substances are released. The left side of Figure 4 shows how liposomes and liposomes that have been changed by polymers are made from natural membrane pieces. This means that they are very similar to biological barriers in terms of their makeup. The right panel shows hydrogel-assisted delivery, which is when a liposome and a recipient membrane come together to make ion channels. This improves the balance of hydration and ionic communication across the wound interface. These processes show that lipid–polymer–hydrogel platforms that work together can speed up the delivery of drugs and help tissues heal. They show how nanoscale assembly principles can be used to improve wound treatments.

#### 3.2.2. Solid Lipid Nanoparticle (SLN) Engineering

SLNs are lipid nanosystems made from natural lipids that are less likely to be harmful and can be made without the use of solvents. They also help active ingredients that do not dissolve well in water enter the skin better and become more available to the body. [83]. Some of their strengths are that they can keep labile cargos safe, are more stable in storage than liposomes, and can handle high drug concentrations and lyophilization [79]. There are still problems, like APIs that take a long time to load, being ejected during polymorphic transitions in storage, and dispersions with a lot of water [84]. Some ways to produce SLNs are HPH, microemulsion, ultrasound, solvent emulsion-evaporation, emulsion-diffusion, membrane contact methods, and SCF extraction with CO_2_. You can use W/O/W to package hydrophilic substances, but it does not stay stable in media that have a lot of enzymes and proteins [85]. When you are ready to decide on characterization, you should include size and PDI by PCS and LD, zeta to estimate storage stability, crystallinity with polymorphic mapping, the presence of secondary colloids, drug content, release in exudate-like media, and surface morphology. Many studies exclusively utilize water-based PCS and singular zeta values. This makes things look more stable than they really are and makes it harder to figure out how much to provide to wounds that have proteases, salts, and reactive oxygen species (ROS) [38]. Batch disclosure, strict limits on residual and endotoxin levels, and changing the product’s form to dry powders by spray-drying or lyophilization all help make translation easier [86].

#### 3.2.3. Nanostructured Lipid Carrier (NLC) Engineering

NLCs blend solid and liquid lipids to create imperfect lattices that raise loading, suppress expulsion during storage, and enable tunable release for cutaneous use. A typical process heats the lipid blend above Tm with cargo, forms a pre-emulsion with aqueous surfactant, then applies HPH or US and controlled cooling to obtain a solid dispersion [87]. The ratios of solid to liquid lipid, surfactant level, cooling rate, and post-processing with extrusion or microfluidics set size, PDI, and EE, while shaping crystallinity and long-term robustness [88]. The advantages over SLNs include higher capacity and better control of matrix order, although polymorphic transitions, oil leakage under shear, and dehydration during dressing changes remain practical risks [82]. Many reports provide DLS size in water and a single zeta value without testing in media that contain albumin, mucin, enzymes, and ROS, which inflates apparent stability and clouds dose planning [89]. As summarized in Table 2, method descriptions that support translation should add number-based size distributions, DSC or XRD for polymorphism, mass balance for loading and recovery, residual and endotoxin limits, and release in exudate-like media linked to re-epithelialization, biofilm control, and comfortable removal [90].

### 3.3. Polymer–Lipid Hybrid Platforms (PLHN)

**Figure 6:**
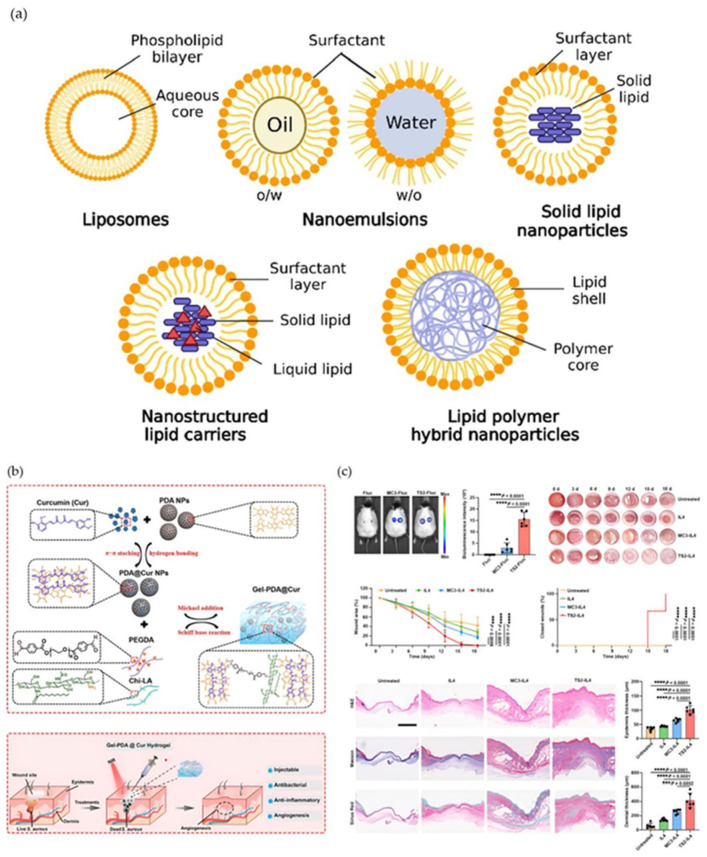
Comparative illustration of lipid nanoparticle-based platforms and their applications in wound repair. (**a**) Structural schematics of major lipid nanoparticle formulations, including liposomes, nanoemulsions, solid lipid nanoparticles (SLNs), nanostructured lipid carriers (NLCs), and lipid–polymer hybrid nanoparticles. (**b**) Representative polymer–lipid–hydrogel hybrid assembly and wound-targeted delivery mechanism illustrating pH-activated PDA–Cur release, PEGDA–CHLA crosslinking, and tissue adhesion properties. (**c**) In vivo wound-healing efficacy and histological analysis demonstrating accelerated wound closure and enhanced regeneration following NLC-based treatment. Black dots indicate wound boundary markers used to delineate the region of interest. Graphs include statistical comparisons. Reproduced with permission from ref. [9]; Copyright 2023, ACS Materials Au.

PLHNs combine a polymer core with a lipid shell to make wound delivery stable and allow the polymer to interact with the membrane [21]. Two-step assembly makes polymer NPs and lipid vesicles separately and then combines them by extrusion, mixing, or US. This lets you tune them separately, but it can lead to uneven coating, uneven surface coverage, and limited scale [99,100]. One-step assembly uses solvent diffusion or emulsion–evaporation to form PLHN directly, and HPH or microfluidics can narrow dispersity, while residual solvent and interfacial defects may trigger the leakage of protease- and salt-rich exudate [85]. Post-insertion of PEG and LbL coatings mitigates aggregation and modifies charge; however, increased corona thickness may impair cell interaction and reduce ligand efficacy [101]. Active targeting often overlooks the effects of ligand orientation loss and the damage inflicted by enzymes and reactive oxygen species in wounds, thereby reducing the advantages in the absence of appropriate controls [102]. Many studies report DLS size in aqueous solutions alongside a single zeta value, providing insufficient information on oxidation, residues, or sterilization effects, leading to uncertainty in dose planning and dressing replacement [103]. Figure 6 schematically compares major lipid nanoparticle systems—liposomes, nanoemulsions, solid lipid nanoparticles, nanostructured lipid carriers, and lipid–polymer hybrids—highlighting how PLHNs merge the structural stability of polymers with the biological adaptability of lipids. Panels (b) and (c) further illustrate a representative polymer–lipid–hydrogel hybrid and its wound healing mechanism, showing sustained release, antioxidative protection, and enhanced tissue regeneration in vivo. Figure 6 contrasts LNP families and locates PLHN among them, emphasizing the need for number-based size distributions, zeta in media, mass balance for drugs and lipids, oxidation and residual limits, and linkage to re-epithelialization, biofilm control, and comfortable removal [9].

### 3.4. Surface Functionalization and Bioactive Loading

**Figure 7:**
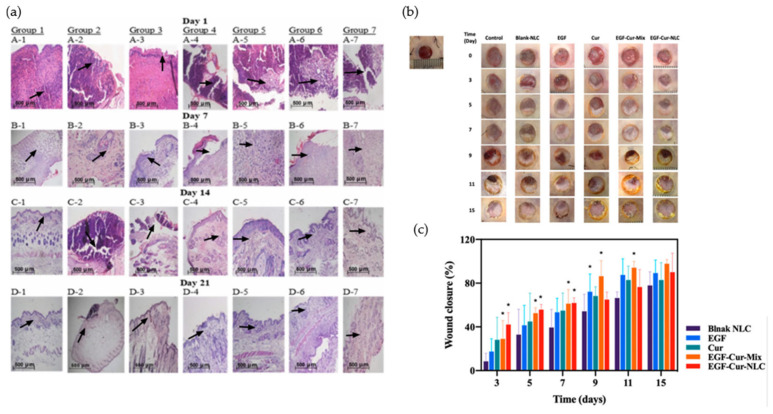
In vivo assessment of wound healing efficacy and histological analysis of EGF–Cur–NLC treatment. (**a**) Typical wound images of diabetic rats treated with Blank NLC, EGF, Cur, EGF–Cur–Mix, and EGF–Cur–NLC formulations over 15 days, showing progressive closure and scab reduction. Arrows indicate key histopathological features including re-epithelialization fronts, granulation tissue formation, and collagen deposition areas. (**b**) Quantitative wound-closure analysis (%) showing that EGF–Cur–NLC had a significantly higher healing rate than other formulations over 15 days. Asterisks (*) denote statistically significant differences compared with the Blank NLC group (* *p* < 0.05). (**c**) Histopathological analysis of skin sections from days 1 to 21 post-treatment, demonstrating re-epithelialization, collagen deposition, and angiogenesis in the EGF–Cur–NLC group. Arrows highlight regenerated dermal structures and newly deposited collagen fibers. Reprinted with permission from Ref. [114]. Copyright 2023, Pharmaceutics.

PLHNs make it easier to deliver drugs directly to wounds by using surface engineering and making loading faster [104]. When you put amphiphilic blocks together, like PLA–PEG, the PEG chains stick out. This makes hydrated coronas that proteins can’t stick to, but ligands can. Once lipid shells are added and covalently linked to polymer cores, peptides, sugars, antibody fragments, or nucleic acids can fit inside. The charge and hydration properties of PLHNs make it easier to deliver drugs directly to wounds by using surface engineering and speeding up the loading process [104]. Changes in PEG’s length and density affect how it holds a charge and how much water it can hold [105]. Double-emulsion and ionizable parts make it easier to put hydrophilic cargo inside endosomes and then release it. On the other hand, mobile bilayers could move ligands around, and dense PEG could keep cells from moving around and interacting with each other inside cells. Exudate has reactive oxygen species (ROS) and proteases that can break apart motifs or hide albumin and mucin epitopes [106,107]. Recent studies (2024–2025) have broadened these concepts to encompass immune-modulatory and stimuli-responsive surface designs. PEG coronas and lipid anchors are progressively integrating ROS- or enzyme-cleavable linkers to coordinate drug release with oxidative or proteolytic stress, thereby improving infection management and wound healing in diabetic individuals [108]. The interfacial chemistry of PLHNs can greatly affect how the immune system works. For example, surfaces that have been changed with arginine-rich or zwitterionic peptides can help M2 macrophages polarize and reduce chronic inflammation [109]. Biomimetic coatings that use macrophage or platelet membranes are even better at moving through the immune system and building up in certain areas [110]. In the method sections, there should be measurements of ligand density and stability, a mass balance for the payload, a list of any leftover reactants, and an analysis of zeta potential and release kinetics in exudate-like media after sterilization and drying. You need this to make a decision [111,112,113]. Figure 7 shows how these strategies for surfaces and formulations affect biological performance. The in vivo wound healing images and quantitative closure data demonstrate that EGF–Cur-loaded NLCs accelerate tissue repair compared to blank or single-agent controls. Histological analyses indicate enhancements in re-epithelialization, collagen deposition, and angiogenesis. These results show that controlled bioactive loading and improved lipid–polymer interfaces have a direct effect on how quickly wounds heal and tissues regenerate [114].

## 4. Therapeutic Functions of Polymer- and Lipid-Based Nanostructures in Wound Healing

### 4.1. Antimicrobial Activity and Anti-Inflammatory Modulation

Microbial loads and persistent cytokine signaling impede the healing process of chronic wounds [115]. Polymer- and lipid-based nanostructures can avoid these problems by keeping the level of treatment the same and making their antimicrobial activity stronger than that of regular dressings. Chitosan–PLGA nanoparticles maintained fibroblast viability by more than 85% while inhibiting the growth of *S. aureus* and *E. coli* within 12 h, demonstrating a strong selectivity between bacterial virulence and cytocompatibility [116]. Chitosan promotes cationic hemostasis and attaches to microbial membranes, enhancing bacterial elimination and thrombus stabilization [117]. SLN, NLC, and lipid–polymer hybrid nanoparticles (LPHN) protect unstable drugs and make it possible to deliver antimicrobial and anti-inflammatory agents in phases during the inflammatory phase [118]. ROS-responsive lipid nanoparticle hydrogels exhibited approximately 85% bacteriostatic efficacy in infected diabetic wounds and decreased TNF-α levels by 70%, facilitating accelerated granulation tissue formation and re-epithelialization by day 7, in contrast to roughly 45% in untreated controls [119]. When fibroblasts are in the proliferator phase, too much exposure to silver or zinc oxide nanoparticles can kill 55–65% of them. This shows how important it is to test with different doses [120]. Cryogels made of gelatin–silver nanoparticles reduced the number of Pseudomonas aeruginosa and Staphylococcus aureus by more than 4 log CFU. However, releasing more than 1 µg mL of silver in vitro caused fibroblast toxicity by 20–30%, which shows that controlled release is necessary. In an albumin-rich medium, hybrid PLGA-lipid nanoparticles kept an effective load of more than 90% for 48 h. They also had a ζ-potential between −10 and 20 mV, which shows that they were stable in colloidal form under conditions similar to exudate [121]. Nanostructures containing both doxycycline and curcumin facilitated M2 macrophage differentiation (M1/M2 ≈ 0.4) and concurrently decreased TNF-α levels by 70% and IL-6 levels by 65%, leading to significant enhancement in granuloma formation and epithelial obstruction in diabetic wounds [122]. Table 3 shows the results of a quantitative comparison of the antibacterial and anti-inflammatory effects of the above treatments at different stages of wound healing. It shows that the polymer- and lipid-based nanostructures work better than regular dressings when it comes to clearing bacteria, lowering cytokines, and speeding up tissue recovery.

### 4.2. Angiogenesis Promotion

**Figure 8:**
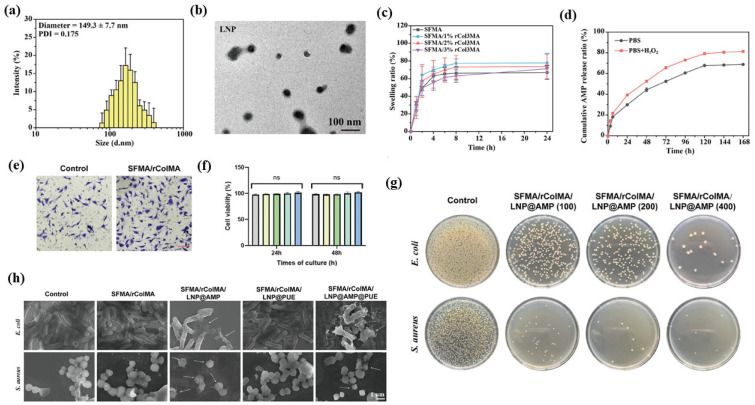
Representative experimental data illustrating multifunctional therapeutic actions of polymer- and lipid-based nanostructures in wound healing. (**a**) Particle size distribution of LNPs (~150 nm, PDI 0.17). (**b**) TEM image confirming spherical morphology. (**c**) Swelling behavior of SFMA/rColMA hydrogels with varying compositions. (**d**) Cumulative AMP release profiles under PBS- and ROS-rich (H_2_O_2_) conditions. (**e**) Fibroblast morphology after incubation on hydrogel surfaces. (**f**) Cell-viability analysis at 24 h and 48 h showing excellent cytocompatibility, with ‘ns’ indicating no significant difference and the bar colors representing each tested formulation. (**g**) Antibacterial activity against *E. coli* and *S. aureus* after treatment with LNP@AMP hydrogels at different concentrations. (**h**) SEM micrographs showing membrane disruption of bacteria after exposure to SFMA/rColMA/LNP@AMP or SFMA/rColMA/LNP@PUE composites, with arrows indicating sites of membrane damage. Reprinted with permission from Ref. [119]. 2024, Advanced Science.

The creation of new microvessels is what makes granulation tissue form and provides nutrients during skin regeneration. Nanostructures made of polymers and lipids can control this process locally by keeping angiogenesis-promoting signals coming in while limiting their spread throughout the body [129]. Liposomes, solid lipid nanoparticles, polymersomes, and polymer–lipid hybrid nanoparticles can deliver vascular endothelial growth factor (VEGF) and basic fibroblast growth factor (bFGF) within a time frame that promotes endothelial cell proliferation, migration, and lumen formation. They can also be used with nitric oxide (NO) donors to improve perfusion in people with diabetes [130,131,132]. The benefits include weak protein protection, co-mounting anti-inflammatory signals, and being close to wet wound surfaces [133]. Recent studies show that VEGF-integrated polymer–lipid and ROS-responsive hydrogel systems greatly improve neovascularization and tissue perfusion in models of chronic and diabetic wounds. By controlling oxidative stress and inflammatory signaling, ROS-removed lipid nanoparticle-based hydrogels help micro-vascularization and re-epithelialization [134]. Dual-growth factor delivery systems, like hydrogels with VEGF/bFGF, release substances that promote angiogenesis for several days. This makes endothelial formation and wound healing much faster than with single-factor systems [135,136]. Figure 8c,d show that lipid nanoparticle hydrogels are controlled by expansion and react with ROS release signals that promote angiogenesis when there is a constant need for them in wound simulation conditions. This constant transmission helps the blood vessels grow back, raises the levels of CD31/α-SMA, and keeps the first capillary network stable [119,134]. Hybrid nitric oxide-releasing liposome hydrogels stimulate HIF-1α and VEGFR2 signaling, promoting angiogenesis and mitigating oxidative stress-induced vascular collapse in diabetic wounds, resulting in increased vascular density and functional recovery [137]. Important caveats remain. Proteases and reactive oxygen species exudate degrade growth factors and destabilize carriers. An uncontrolled burst may lead to the formation of leaky immature vessels [138]. Excess nitric oxide can harm cells through the formation of peroxynitrite, and light-activated donors may be ineffective in penetrating dressings [139]. Many studies persist in performing tests in water at single time points, resulting in an overestimation of stability and obscuring dosing intervals [140]. Study designs must quantify spatial and temporal release in wound-mimicking media and correlate these profiles with capillary density, perfusion imaging, re-epithelialization, and scar quality [141].

For wounds to heal, the formation and remodeling of the extracellular matrix must be coordinated. Polymer and lipid nanostructures give structural cues and control the activity of fibroblasts to make these processes more effective [142,143]. Electrospun nanofiber scaffolds made from biocompatible polymers help fibroblasts stick to each other, move around, and make collagen fibrils. Gelatin nanofibers have arginine–glycine–aspartic acid motifs that help integrins stick to them and integrate with tissue more effectively [144]. Hybrid dressings that mix nanofibers and lipid nanoparticles make it possible to deliver pro-regenerative agents directly to the right place while keeping the area moist, which is good for matrix assembly [145]. Gene-regulatory strategies, such as the administration of small interfering RNA aimed at transforming growth factor beta one, can inhibit excessive collagen accumulation and mitigate hypertrophic characteristics, provided that the dosing is calibrated to prevent the suppression of normal remodeling processes [146]. A recent study has shown that polymer–lipid composite nanofiber scaffolds greatly improve the alignment of collagen and the strength of tissue during remodeling. Electrospun PCL–collagen scaffolds exhibit superior extracellular matrix (ECM) organization and tensile strength compared to single-component systems, promoting expedited substrate maturation and fibroblast infiltration in vivo [147,148]. Double-transmission scaffolds that release TGF-β1 target both small interference RNA (siRNA) and VEGF at the same time have been shown to effectively block signals that cause fibrosis, bring the collagen I/III ratio back to normal, and speed up the rebuilding of the extracellular matrix. This greatly reduces scar thickness and improves tissue structure. These gene-activated constructs integrate anti-fibroblast and angiogenesis-promoting signals within a single platform, enabling time-coordinated regulation of fibroblast activity and vascular regeneration for scarless wound healing [149]. Figure 8e,f show that fibroblasts grown on SFMA/rColMA hybrid hydrogels spread out a lot and had well-defined actin cytoskeletons. They formed thick, straight collagen fibers in less than 48 h. Fluorescence staining for life and death showed that more than 90% of the cells were alive, and confocal imaging showed that the ECM structure was uniform in three dimensions, which meant that the cells were strongly interacting with the substrate and were very biocompatible [150]. Mechanically, these hybrid nanostructures regulate cytoskeletal tension and substrate reconstruction by activating the focal adhesion kinase (FAK) and integrin-associated kinase (ILK) signaling pathways. This regulation encourages fibroblasts to change into a phenotype that promotes regeneration, helps keep collagen I/III levels balanced, and stops excessive crosslinking and stiffness, which are common signs of fibrosis [151]. Important risks remain. Proteinases and reactive oxygen species in wound fluid can degrade bioactive payloads and weaken scaffold mechanics, residual crosslinkers or solvents may irritate the peri-wound margin, and overly adhesive matrices can hinder cell infiltration and removal [152]. Many studies assess fibers and carriers in water at single time points, leading to an overestimation of stability and a lack of clarity regarding dose schedules [153]. Development should quantify release and mechanical recovery in wound-mimicking media and relate these profiles to collagen architecture, re-epithelialization, and scar quality [141].

### 4.3. Targeted and Stimuli-Responsive Growth Factor Delivery

Polymer and lipid nanostructures enable spatiotemporal control in wound therapy via local sensing and on-demand release, reducing off-site exposure [154]. Smart dressings that monitor acidity or temperature can initiate antibiotic release from temperature-responsive hydrogels, sustaining effective levels during initial contamination without the need for repeated handling of delicate tissue [155,156]. Liposomes, solid lipid nanoparticles, polymersomes, and polymer–lipid hybrid nanoparticles can present vascular endothelial growth factor and basic fibroblast growth factor in sustained or pulsed modes that support endothelial migration and perfusion, and carriers that liberate nitric oxide can improve microcirculatory flow in ischemic beds [12,157]. A recent study has demonstrated that polymeric–lipid hybrid hydrogels responsive to pH and reactive oxygen species (ROS) can sustain VEGF-dependent release profiles and markedly enhance local angiogenesis compared to non-responsive systems [158]. The more responsive chitosan–lipid nanocomposites were also designed to induce antimicrobial or antibiotic release under mild high heat (about 37 to 40 °C), resulting in improved bacteriostatic effects and faster wound sutures compared to passive hydrogels [159]. A dual-stimulation platform incorporating near-infrared (NIR) and redox/ROS triggers enables the sequential release of VEGF and nitric oxide (NO), promoting increased blood flow and stable neovascular network formation in chronic wound models [160,161]. These observations demonstrate the ability to deliver on demand, consistent with the pH- and ROS-induced antimicrobial peptide (AMP) release profiles (Figure 8d) observed in LNP hydrogels. SEM images (Figure 8h) further show bacterial membrane breakdown, including pore formation and surface collapse, which supports catalytic and oxidative bactericidal action [162]. Design tradeoffs require early attention because proteases and reactive oxygen species in exudate degrade proteins and destabilize carriers, uncontrolled bursts can generate immature leaky vessels, excessive nitric oxide forms cytotoxic species, and sensor drift or poor light penetration can misfire triggers [163]. Laboratory protocols that rely on simple buffers and single time points overstate stability and obscure dosing intervals. Future evaluations should quantify release half-life and trigger responsiveness in exudate-mimicking media and correlate these with perfusion index, biofilm suppression rate, and scar thickness reduction to support translational reproducibility.

## 5. Polymeric Platforms and Hydrogel Composites for Wound Healing

### 5.1. Natural Polymer Platforms

**Figure 9:**
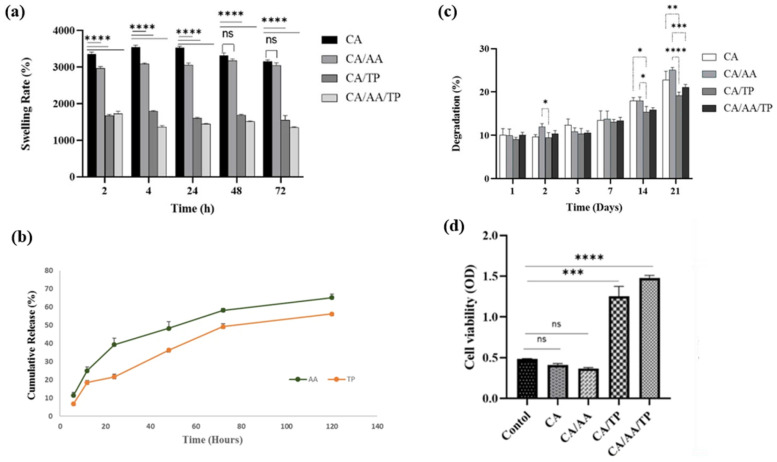
Quantitative characterization of chitosan–alginate composite hydrogels incorporating ascorbic acid (AA) and tannic acid (TP). (**a**) Swelling behavior showing rapid hydration within 2 h and reduced water uptake in AA/TP-modified hydrogels. (**b**) Cumulative release profiles of AA and TP demonstrating sustained release over 120 h. (**c**) Controlled biodegradation profile indicating moderate mass loss (≤40%) over 21 days. (**d**) Cell viability (MTT assay) confirming excellent cytocompatibility of AA/TP formulations relative to control groups. Statistical significance indicators: * *p* < 0.05, ** *p* < 0.01, *** *p* < 0.001, **** *p* < 0.0001, ns = not significant. Reprinted with permission from Ref. [164]. 2023, BMC.

Chitosan, gelatin, and alginate exhibit hemostatic, antibacterial, moisture-retentive, and extracellular matrix-guiding properties for localized therapeutic use in wound healing polymers [164]. Natural polysaccharides have different charges, molecular structures, and degradation kinetics, which determine their wound-specific applicability and healing performance. Chitosan exhibits strong antibacterial and hemostatic properties thanks to its cationic amino groups, which promote thrombus formation and macrophage activation to achieve wound closure rates of approximately 85 to 90% and bacterial inhibition rates of over 90% within 10 days in infected wound models [165,166]. Alginate creates a hydrogel by exchanging Ca^2+^ and Na^+^ ions with wound exudate. It keeps moisture in at a rate of about 85 to 90% and closes at a rate of about 90% after 10 days. But too much crosslinking by calcium can cause too much gelation and make the material weaker [167]. Fucoidan, a sulfurized polysaccharide obtained from brown algae, enhances antioxidant and angiogenic activity by upregulating VEGF and inhibiting ROS. In ischemic or diabetic wound models, the fucoidan-based hydrogel achieves a suture rate of approximately 93% and a reduction of approximately 40% of active oxygen species (ROS) within 10 days [168,169]. Chitosan, alginic, and fucoidan complexes produce porous and compressible scaffolds and exhibit a variety of antimicrobial and angiogenic properties. In anterior wounds, these hybrid materials achieved a closure rate of approximately 95% and a collagen I/III ratio approximately 1.8-fold higher within 10 days, indicating improvements in angiogenesis and extracellular matrix restructuring [170]. Figure 9a–d show that the antioxidant-embedded chitosan–alginate hydrogel rapidly expanded, was continuously released for more than 48 h, degraded in a controlled manner, and kept the cells alive for more than 95% of the time. This indicates that the cells were suitable and stable during the wound healing process [164].

Table 4 further summarizes the representative polymeric materials discussed in this section, highlighting how their structural differences, advantages, limitations, and quantitative healing efficiencies contribute to distinct wound healing mechanisms.

### 5.2. Synthetic Biodegradable Polymer Platforms

Controlling molecular weight, erosion rate, and mechanical properties through synthetic biodegradable polymers lets you customize wound healing nanostructures for local treatment delivery [82]. The representative synthetic biodegradable polymers poly (lactic-co-glycolic acid) (PLGA), polycaprolactone (PCL), and polyethylene glycol (PEG) provide adjustable degradation rates, mechanical stability, and controlled drug release properties, making them widely utilized as scaffolding materials for wound healing applications [183]. PLGA effectively encapsulates antibiotics, anti-inflammatory drugs, proteins, and nucleic acids. Its hydrolysis gradually releases glycolic and lactic acids, which help new blood vessels grow and rebuild collagen matrices in diabetic and burn wound models [184]. The improved PLGA scaffold closed more than 90% of wounds in 10 days and also sped up granulation and re-epithelialization. This shows that it could be helpful for treating chronic wounds [185]. But to keep the pH level stable during the process, researchers often use PEG mixing or a calcium carbonate buffer. This is because acid decomposition products can irritate the tissues around the wound or make sensitive biomolecules less stable [184]. PCL is a semi-crystalline polymer that slowly dissolves and provides strength and long-term stability. It makes an electrospinning mesh that sticks to uneven wound sites and keeps its shape for up to four weeks [186]. In vivo studies showed a wound closure rate of about 85–90% and a fibroblast proliferation rate of about twice that of the PLGA scaffold, suggesting the mechanical superiority and bio-compatibility of PCL [180]. However, PCL’s high crystallinity and hydrophobicity can make it harder to break down and cause it to release quickly during the first hydration process. Therefore, surface bonding using hydrophilic PEG chains or adhesive peptides was introduced for the improvement of wettability and cell adhesion [96]. E.g., a hydrophilic polymer and surface modifier improves protein stability, solubility, and bio-compatibility while reducing nonspecific protein adsorption [17]. PEG-based hydrogels maintain a moisture retention rate of 90 to 95% under exudate-like conditions, support fibroblast survival rates of over 90%, and reduce bacterial loads by approximately 70% [187]. However, dense PEG corona can inhibit cell adhesion, and although immune recognition for PEG is rare, molecular weight and purity must be carefully managed [188].

### 5.3. Hydrogel Nanocomposites and Films

**Figure 10:**
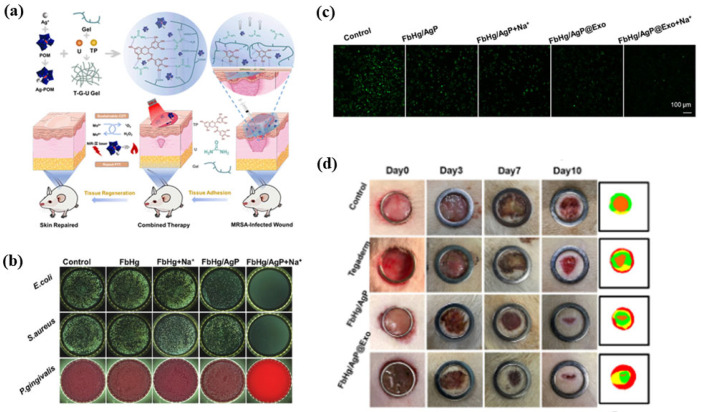
Multifunctional Ag–polyoxometalate (Ag-POM) hydrogel nanocomposites for infected-wound healing. (**a**) Schematic of Ag-POM/gelatin–urea hydrogel showing catalytic and photothermal antibacterial effects. (**b**) Antibacterial activity against *E. coli*, *S. aureus*, and *P. gingivalis*. (**c**) Live/dead fluorescence images of bacteria after hydrogel treatments. (**d**) Wound-closure images over 10 days showing markedly accelerated healing in the FbHg/AgP@Exo and FbHg/AgP@Exo + Na⁺ treatment groups. The color-coded maps on the right visualize wound-area quantification, where green represents the initial wound boundary, yellow indicates intermediate healing, and red denotes the remaining unhealed area. Reprinted with permission from Ref. [190]. 2022, Elsevier.

Hydrogel nanocomposites combine water-rich polymer networks with therapeutic nanoparticles to heal wounds, control moisture, and give medications at the right time and place [189]. Crosslinked matrices such as gelatin, chitosan, or alginate facilitate conformal contact and elasticity, whereas embedded carriers, including liposomes, silver polyoxometalate clusters, and plant-derived polyphenols, promote antimicrobial activity, near-infrared photothermal heating, and pro-regenerative signals [190,191]. A representative data integration hydrogel system is the GelMA–liposome hybrid matrix, which double-encapsulates small molecules and growth factors to achieve a phased release of peptides and continuous fluorescence expression in vivo while maintaining expansion ability and degradation stability [192]. This hybrid structure allows for spatial and temporal control of transmission and local regeneration signaling, which improves the balance of tissue integrity and angiogenesis in wound environments [193]. Complementary, multifunctional Ag-polyoxometalate (Ag-POM) hydrogels combine catalytic antimicrobial chemistry with mild photothermal effects, effectively inhibiting methicillin-resistant S.aureus (MRSA) and accelerating collagen deposition and epithelial regeneration in whole-layer wound models (Figure 10a–d) [190]. As shown in Figure 10a, the Ag-POM/cha polyphenol/gelatin hybrid hydrogel possesses a modular design that integrates catalytically active oxygen (ROS) regulation and near-infrared (NIR) reactive photothermal sterilization activity. Figure 10b shows a wide range of antimicrobial effects against *E. coli*, *S. aureus*, and *P. gingivalis*, achieving a bacterial reduction rate of >95% in 6 h. Life-and-death fluorescence imaging (Figure 10c) demonstrates efficient bacterial membrane destruction while minimizing fibroblast toxicity (<10%), confirming the biosafety profile [194]. In vivo wound healing experiments (Figure 10d) demonstrate rapid epithelialization, attaining a minimum of 85% healing by day 7, accompanied by organized collagen alignment. This shows how catalytic free oxygen regulation and local photothermal treatment work together to make things better. These results suggest that the combination of catalytic and photothermal properties in the polymeric hydrogel matrix may improve wound healing while reducing excessive thermal accumulation or cytotoxic stress. For clinical application, subsequent studies are required to validate manufacturing stability and biological safety via reproducible degradation kinetics, quantified release profiles, perfusion recovery mapping, and stability assessment of post-sterilization crosslinking agents [188].

## 6. Lipid-Based Nanostructure Platforms

### 6.1. Conventional and Deformable Liposome Platforms

**Figure 11:**
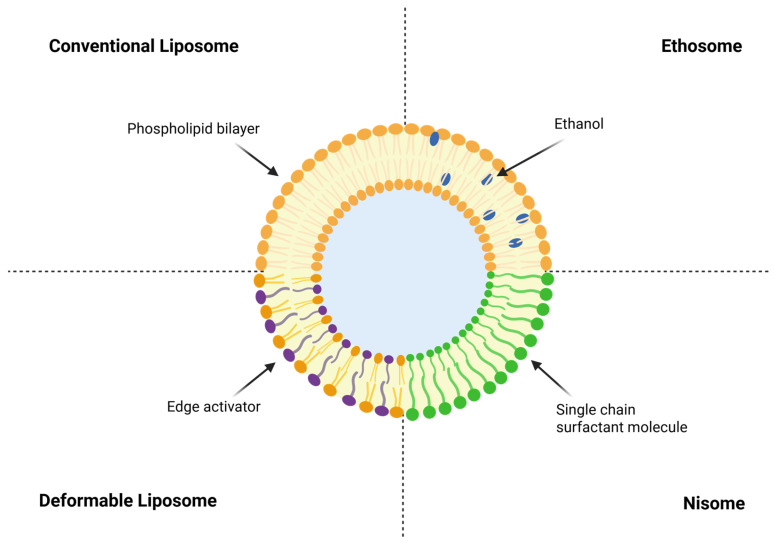
Schematic representation of liposomal architecture for topical and transdermal delivery. Conventional liposomes consist of a phospholipid bilayer surrounding an aqueous core. Deformable liposomes incorporate edge activators, such as sodium cholate or polysorbates, that enhance bilayer fluidity and facilitate passage through narrow pores. Ethosomes contain ethanol within the bilayer, which increases membrane permeability and improves skin penetration. Niosomes are formed from nonionic surfactants, providing a phospholipid-free and chemically stable alternative.

Liposomes are bilayer vesicles that entrap hydrophilic agents in an aqueous core while accommodating lipophilic agents within the membrane, and the membrane composition echoes the stratum corneum, which supports topical delivery [195]. Conventional liposomes frequently stall within superficial layers and lose integrity in protein-rich exudate, which limits access to the dermis and promotes premature leakage [196]. Existing liposomes allow for controlled release, but they do not always penetrate the skin well and are less stable in protein-rich exudate. This can cause drugs to leak out too soon and settle on the surface of the skin [197]. Albumin adsorption and membrane instability usually hurt the integrity of liposomes in physiological conditions, which makes them less bioavailable [198]. To overcome these limitations, modifiable liposomes have been developed to improve bilayer elasticity and hydration sensitivity by incorporating modification activators like sodium cholate, Tween 80, and Span 80 [199]. This flexibility lets it go deeper into the surviving epidermis and dermis than regular liposomes, which makes it easier for drugs to penetrate the skin and wounds without changing the surface morphology too much. But too much surfactant can cause stimulation and make encapsulated biomolecules unstable. This shows how important it is to have the right formulations. Ethosomes that add ethanol to the phospholipid bilayer make the membrane more fluid and the lipids more soluble, which makes the skin more permeable. Formulations with 20–40% ethanol had drug flow rates that were dozens of times higher than those of regular liposomes. However, they can make sensitive skin dry or irritated for a short time [198]. Niosomes are made of nonionic surfactants instead of phospholipids. This makes them more stable and less expensive, and they keep drugs encapsulated well even after long periods of storage [197]. Epidermal penetration depth generally reaches a viable epidermal layer (100–200 µm) depending on surfactant type and vesicular elasticity, but cytotoxicity and protein adsorption vary with formulation [200]. Overall, modifiable ethanol-based nonionic vesicles provide a versatile strategy for improving local and transdermal delivery of antimicrobial and anti-inflammatory drugs while maintaining biocompatibility and structural stability under exudate-like conditions. Figure 11 summarizes these architectures and highlights the need for decision-ready characterization that reports number-based size distributions and surface charge in exudate-like media, monitors lipid oxidation, verifies stability after sterilization and drying, and links transport to outcomes that include biofilm penetration, re-epithelialization, and comfort on removal.

### 6.2. Solid Lipid Nanoparticles and Nanostructured Lipid Carrier Platforms

**Figure 12:**
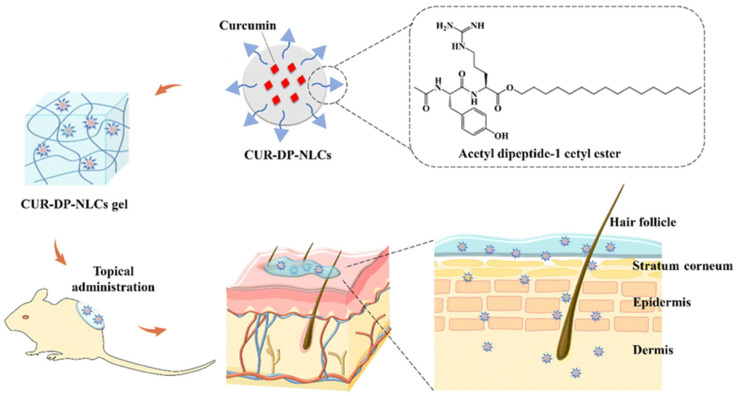
Schematic representation of curcumin-loaded nanostructured lipid carriers (CUR-DP-NLCs) incorporated into a gel system for topical wound therapy. The CUR-DP-NLCs, stabilized with acetyl dipeptide-1 cetyl ester, enhance curcumin encapsulation and stability. Following topical administration, the formulation penetrates the stratum corneum and distributes through the epidermis into the dermis, enabling sustained curcumin delivery. This process promotes anti-inflammatory and antioxidant effects, reduces bacterial colonization, and accelerates tissue regeneration for effective wound healing. Reprinted with permission from ref. [174]; 2023, RSC Advances.

SLNs provide a biocompatible solid lipid matrix that protects unstable active ingredients and allows for long-term use on wound surfaces and follicles [201]. SLN is a matrix that is safe for living things and can be used to deliver drugs through the skin. However, the structure can change when crystals are stored, which can cause internal molecules to leak over time. This can compromise the stability of the encapsulated active ingredient [202]. Hydration cycle and mechanical shear force can also cause rapid release, which leads to a decrease in long-term exposure effects in conditions such as wound exudate [203]. In simulated exudate media containing albumin, standard SLNs typically exhibit significant drug leakage during the first 12 to 24 h, implying limited colloidal stability under physiological stress [204]. NLC addresses certain limitations by generating lattice imperfections through the amalgamation of solid and liquid lipids. This increases drug loading capacity and prevents excretion during the crystallization process. When you change the solid-to-liquid lipid ratio (about 70:30 *w*/*w*) and the surfactant concentration, you usually obtain a 1.3 to 1.8 times better encapsulation efficiency and a longer release time (t_1_/_2_ ≈ 20 to 30 h) than SLNs [205]. The release kinetics, particle size (100–180 nm), and polydispersity (<0.3) are very dependent on the cooling rate and lipid composition, which affects how stable the material is mechanically and chemically [202]. In a full-layer mouse model, NLC with curcumin and asiaticosides sped up wound healing, with a closure rate of about 85–90% within 12–14 days compared to controls. It also reduced reactive oxygen species by about 30–40% and increased collagen deposition [206]. These therapeutic enhancements stem from the synergies of continuous drug release, antioxidant activity, and the regulation of inflammatory signaling. Nevertheless, key challenges to be addressed in future formulation designs remain emulsion migration, polymorphic conversion, surfactant-associated stimulation, and instability under enzyme- or salt-rich exudate. Figure 12 illustrates a curcumin-loaded nanostructured lipid carrier gel stabilized by acetyl dipeptide-1 cetyl ester that traverses the stratum corneum and distributes through the epidermis into the dermis for sustained delivery, which is promising for antioxidant and anti-inflammatory support but still requires dose and safety verification under wound-relevant conditions. Future development should focus on verifying dose safety and post-sterilization stability under wound-relevant hydration cycles [207].

### 6.3. Nonlamellar Lipid Mesophase Platforms

Cubosomes and hexosomes are lipid particles that are not lamellar. They have water and lipid channels that connect them, which allows hydrophilic and hydrophobic agents to be loaded at the same time. They also protect fragile peptides from being broken down by enzymes in exudate [208]. Bicontinuous cubic structures promote rapid hydration and possess extensive interfacial areas that enhance cutaneous distribution, whereas inverse hexagonal structures frequently impede outward diffusion and are desirable for prolonged release in dressings [209]. Cubosomes and hexosomes are non-structured lyotropic liquid crystal nanoparticles, suitable for loading various cargoes and protecting unstable active ingredients by forming aqueous–lipid pathways in which double continuous cubic or inverted hexagonal lattices are interconnected [208]. Monoolein-poloxamer cubosome formulations demonstrate the acceleration of wound healing in rodent skin models, supporting their usefulness as skin drug carriers, but the reported closure rates vary with drug loading and model [210]. Hexosome (HII) systems provide more continuous diffusion-restricted release than the cubic phase due to their narrow channel and phase-dependent transport properties, and a cubic ↔ hexagonal phase transition within the physiological range has been reported [211]. In vivo experiments on liquid crystal gels (LLCs) in chronic wound models have demonstrated that the incorporation of bioactive factors expedites wound healing, enhances collagen deposition, and diminishes inflammatory infiltration. Steps like drying, lyophilizing, or sterilizing can change the lattice spacing, which in turn changes the release kinetics and reproducibility [212]. For translation purposes, subsequent reports must encompass particle size, lattice constant, and release performance during the wound-mimicking hydration cycle, which is associated with epithelialization, collagen alignment, and removal efficiency [213]. In general, well-optimized cubic and hexagonal mesophases allow for controllable continuous delivery and are good for the skin, which is pushing this field toward structure–activity-based design instead of qualitative statements.

### 6.4. Lipid–Polymer Hybrid Vesicle Platforms

**Figure 13:**
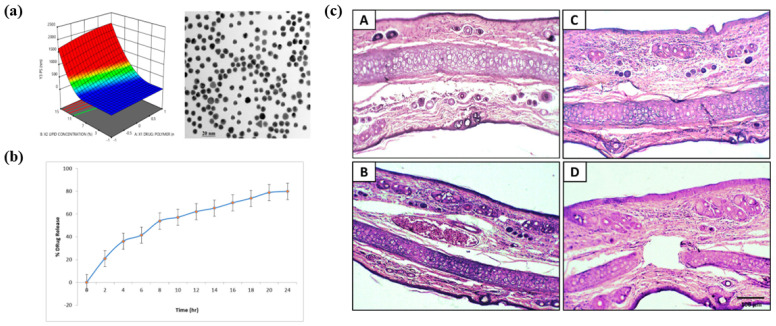
Characterization, release behavior, and skin tolerance of hydrocortisone-loaded lipid–polymer hybrid nanoparticles (HCL-LPHNs). (**a**) 3D response surface plot and TEM image showing uniform spherical nanoparticles within the optimized size range. The color gradient in the surface plot represents changes in particle size across the tested formulation variables. (**b**) Cumulative drug-release profile indicating sustained release of HCL over 24 h. (**c**) Histological sections (**A**–**D**) of rat skin after topical application of L-PHHN formulations showing intact epidermal and dermal layers without significant irritation or inflammatory changes. Reprinted with permission from Ref. [215]. 2023, ACS Publications.

Lipid–polymer hybrid vesicles (LPHVs) are the best way to treat local wounds because they combine the biomimetic interfacial behavior of lipids with the mechanical stability and controllable degradation properties of polymers [17]. These hybrid nanostructures create a stable bilayer or core–shell shape, which allows for long-term colloidal stability and controlled drug release (Figure 13a–c). As shown in Figure 10a, the optimized LPHV formulation produces uniform spherical nanoparticles (~150 nm) with ζ-potential in the range of −10 to −25 mV, maintaining structural integrity even under periodic hydration and shear [214]. Drug release analysis (Figure 13b) confirms stable encapsulation and progressive effective load transfer under simulated exudate conditions, showing that they are released without continuous diffusion and burst effects for 24 h [215]. Histological evaluation (Figure 13c(A–D)) indicates that LPHV dressing maintains the epidermis and dermis without inducing inflammation or irritation in vivo, thereby demonstrating enhanced safety and biocompatibility in whole-layer wound models [17]. For clinical applications, manufacturing methods like solvent diffusion, emulsion evaporation, and microfluidic mixing should be improved to ensure that residual solvent removal, sterilization stability, and batch reproducibility are all good [12]. A thorough characteristic analysis must encompass particle size (100–200 nm), ζ-potential (−10–25 mV), encapsulation efficiency, and the determination of half-life of emission in exudate-like media, which should correlate with biofilm inhibition, reepithelialization, and angiogenesis outcomes to establish a solid structure–function relationship [215].

## 7. Preclinical-to-Clinical Translation and Scalable Production of Smart Wound Dressings

### 7.1. Preclinical Model Systems for Wound Healing Nanostructure Evaluation

Preclinical model systems help establish the translational value of polymer- and lipid-based nanostructures for wound healing [216,217]. However, common rodent excisional wounds close primarily by contraction and underrepresent ischemia, neuropathy, and polymicrobial biofilm that shape human chronic ulcers, constraining inference [218]. Three-dimensional human skin equivalents with stratified epidermis and dermis, organotypic models that integrate vascular and immune components, ex vivo human skin under controlled perfusion and infection, and porcine full-thickness wounds more faithfully capture epidermal architecture, adnexal structures, and biomechanics [219,220]. Chronic wound surrogates that incorporate protease-rich exudate, reactive oxygen species, recurrent bacterial challenge, impaired perfusion, diabetes, and pressure-related tissue deformation test nanostructure stability, cargo retention, release kinetics, and biofilm control [221].

### 7.2. Manufacturing, Sterilization, and Scalability

How useful polymer- and lipid-based nanostructures are for treating wounds depends on how scalable they are to make. The size, shape, charge, and release properties of particles are mostly affected by the temperature, the ratio of solvent to solid, the amount of energy added, and the order in which the particles are mixed [222]. Continuous microfluidic mixing gives you precise control and a narrow size range, but you need materials that will not dissolve in the solvent and cleaning methods that have been tested [223]. It is easy to scale up with high-pressure homogenization and wet grinding, but shear and heat need to be controlled to keep the material from breaking down [224]. When combined with automatic feedback control, process analysis techniques (PATs) that use real-time particle size measurements and spectroscopy can help keep quality stable [225]. Sterilization and stability are still big problems. Most nanostructures are ruined by steam and filtration processes. To keep them from clumping together again, freeze-drying with cryoprotective agents like sucrose or trehalose is the best method [226,227]. The cost and purity of the materials also make it hard to sell, which shows how important it is to have an energy-efficient continuous system that follows the rules for solvent recovery and Good Manufacturing Practices (GMP) [228,229,230]. To keep quality high on a large scale, future manufacturing should use closed continuous lines, AI-based supervision, and sensors that have been tested [231].

### 7.3. Smart and Personalized Wound-Dressing Systems

**Figure 14:**
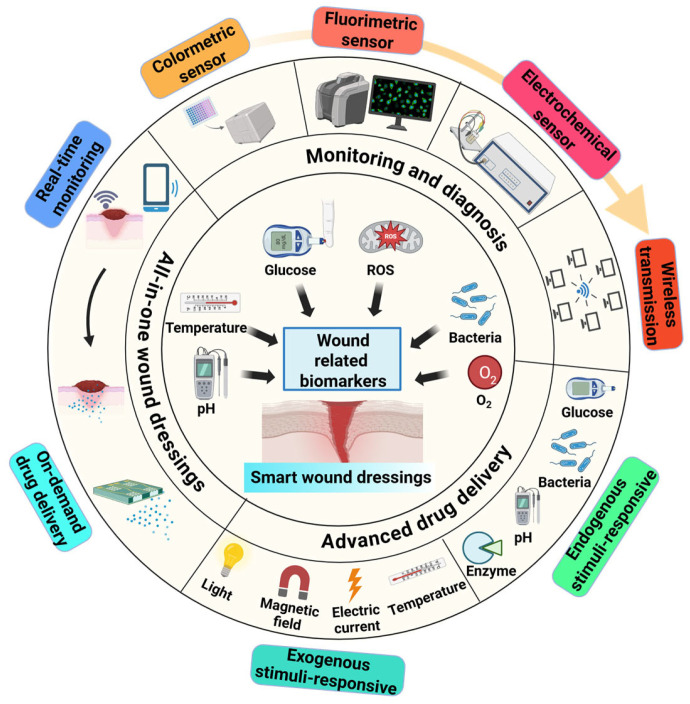
Concept of smart wound dressings that integrate colorimetric, fluorometric, and electrochemical sensors for real-time monitoring of temperature, acidity, glucose, oxygen, bacterial burden, and reactive oxygen species with wireless transmission. Polymer and lipid nanostructures enable endogenous stimulus responsiveness to acidity, enzymes, and bacterial metabolites, and permit exogenous control by light, magnetic field, electric current, and controlled warming for on-demand therapy. The platform illustrates all-in-one wound management that links monitoring and early diagnosis with personalized drug delivery.

Smart wound-dressing systems combine polymer and lipid nanostructures with multiple types of sensors, wireless communication, and stimulus-responsive release to make sure that treatment matches the changing biology of the wound [232,233]. Colorimetric, fluorometric, and electrochemical components measure temperature, acidity, glucose, oxygen, bacterial load, reactive oxygen species, and inflammatory cytokines like interleukin-6 and interleukin-10 [234]. Wireless modules send data to patient devices, and heaters, soft electrodes, microneedles, and microfluidic reservoirs give antimicrobials, anti-inflammatory agents, or electrical stimuli when the sensor thresholds are met [234]. Nanostructures that are tuned to internal signals respond to acidity, enzymes, and bacterial metabolites. External signals include light, magnetic fields, electrical current, and controlled warming [235]. Personalization occurs when artificial intelligence analyzes Internet of Things data streams and clinical images to modify dosage and timing. Additionally, three-dimensional printing facilitates the creation of patient-specific geometries using bioinks that incorporate patient-derived cells or factors [236]. The promise is faster closure, less scarring, and fewer visits to the doctor, but calibration in protein-rich and salt-rich exudate, mechanical durability under bending, gentle adhesion that protects periwound skin, reliable power management with safe disposal, and clear data governance for home care are still needed [237]. Figure 14 shows the general idea and functional integration of these smart wound-dressing systems. It shows how polymer- and lipid-based nanostructures can monitor wound-related biomarkers in real time and release drugs in response to stimuli.

## 8. Conclusions

Polymer- and lipid-based nanostructures present a practical pathway to overcoming the limitations of conventional wound dressing by maintaining local exposure, regulating moisture, reducing microbial load, and inducing angiogenesis and extracellular matrix organization. The evidence summarized in this review highlights the recurring risks that need to be addressed prior to extensive clinical applications, including excessive dependence on aqueous properties, instability in proteases and salt-rich exudates, decomposition of active ingredients during sterilization or drying processes, ligand obstruction in protein body fluids, and dependence on rodent wounds that heal through contraction rather than reepithelialization. Progress will be stronger when development links ex vivo human skin and porcine models with predefined, patient-centered outcomes such as time to closure, pain on removal, quality of the regenerated tissue, and reduction in biofilm burden, while reporting number-based size distributions, surface charge in wound-like media, oxidation markers, residual solvent profiles, and post-sterilization performance. Continuous mixing, process analytical technologies, and verified cleaning and containment can all help with scalable manufacturing. It is also important to think about the cost, supply security, and environmental impact of single-use parts early on. In the future, hybrid nanostructures ought to develop into multipurpose smart dressings that integrate wireless data transmission, adaptive drug release, and real-time biosensing. By combining polymer–lipid platforms with the Internet of Things and AI-driven analytics, it may be possible to remotely monitor infection biomarkers, pH, and temperature, directing on-demand therapeutic release with little patient involvement. Furthermore, in order to balance clinical effectiveness with environmental responsibility and make sure that advanced wound care is in line with the principles of circular healthcare, it will be essential to design sustainable, bio-derived, and recyclable materials. Smart dressings that use nanostructures, the Internet of Things, and artificial intelligence (AI) can personalize care through monitored triggers and responsive release, provided that calibration in complex exudate, gentle adhesion at fragile margins, reliable low-profile power, safe disposal, and strong data governance are in place. In the short term, the focus should be on strong formulations that clearly help with re-epithelialization, infection control, vascularization, and scar quality. After that, targeted clinical trials should be performed to compare performance to current standards while being open about methods and materials.

## Figures and Tables

**Table 1:** Comparative physicochemical and biological features of polymer-, lipid-, and polymer–lipid hybrid-based nanocarriers for wound healing. Summarized properties include particle size control, biocompatibility, tissue adhesion, degradation and release profile, surface functionalization options, morphology and stability, and wound healing performance.

Property	Polymer-Based	Lipid-Based	Polymer–Lipid Hybrid	Ref.
Size control	Broad tunability via polymer type and fabrication process.	Governed by lipid composition and process parameters	Adjustable by polymer–lipid ratio and co-assembly technique	[9,17]
Biocompatibility	High with degradable polymers (PLGA, PEG, chitosan)	Excellent due to membrane-mimetic structure	Combined stability and compatibility	[10]
Tissue adhesion	Strong with mucoadhesive polymers	Moderate, improvable with ligands or PEG derivatives	Tunable via core–shell chemistry	[11]
Degradation and release	Controlled by polymer erosion rate	Diffusion across lipid bilayers	Sequential release from shell and core	[12]
Surface functionalization	Wide range of PEGylation/ligand conjugation	Ligand anchoring within bi-layer; ionizable lipids for pH response.	Dual functionalization across core and shell.	[13]
Stability and morphology	Rigid, low polymorphism risk	Sensitive to phase transition/serum instability	Core stability with bilayer versatility.	[14]
Advantages in wound care	Sustained release, mechanical durability, barrier maintenance	Excellent biocompatibility, conformal contact	Dual loading capacity, balanced penetration, and residence	[17]
Practical limitations	Acidic degradation products, burst risk	Structural instability, lipid polymorphism	Manufacturing complexity, sterilization sensitivity	[12]

**Table 2:** Fabrication strategies for polymer, lipid, and hybrid nanostructures used in wound healing. Columns list category, technique, principle, advantages, and limitations. Rows include PNP via nanoprecipitation and emulsion–solvent evaporation, SLN via HPH, microemulsion, ultrasound, NLC via melt emulsification with controlled cooling, and PLHN via one-step assembly and two-step coating with microfluidics or HPH.

**Category**	**Technique**	**Principle**	**Advantages**	**Limitations**	**Ref.**
Polymer based	Nanoprecipitation	Solvent diffusion and polymer nucleation.	Mild, narrow size, scalable.	Low hydrophilic loading, solvent residue.	[12]
	Emulsion–solvent evaporation (O/W or W/O/W)	Polymer solution in emulsion, solvent removal.	Versatile, tunable size.	Solvent residue, batch variation.	[91]
	Electrospinning	High-voltage jet to nanofibers.	High area, sustained release.	Low yield, burst release.	[92]
Lipid based	SLN (HPH/microemulsion)	Melt dispersion and solidification.	Biocompatible, stable, deep delivery.	Drug expulsion, limited load.	[93,94]
	NLC (melt emulsification)	Solid–liquid lipid lattice formation.	High load, controlled release.	Oil leakage, shear sensitivity.	[95,96]
	Liposomes (TFH/EI/REV/DR/HPH)	Self-assembled bilayers.	Dual loading, surface modifiable	Oxidation, leakage.	[97]
Hybrid	PLHN one-step assembly	Polymer–lipid co-assembly.	Single-step, tunable core–shell.	Solvent residue, leakage.	[98]
	PLHN two-step coating	Polymer NP coated with lipid vesicle.	Independent core/shell control.	Patchy coating, low reproducibility.	[12]

**Table 3:** Quantitative performance of polymer- and lipid-based nanostructures in antibacterial and anti-inflammatory wound healing.

**Stage of Wound Healing**	**Key Events**	**Limitations of Conventional Dressings**	**Performance of Polymer- and Lipid-Based Nanostructures**	**Ref.**
Hemostasis	Platelet activation, fibrin network formation, clot stabilization	Passive protection, delayed clotting, insufficient hemostasis	Rapid hemostasis, reduced clotting time (5 min → 2 min), decreased blood loss (≈45%)	[117,123,124]
Inflammation	Neutrophil recruitment, macrophage activation, bacterial clearance	Weak antibacterial activity, excessive cytokine release	Enhanced bacterial inhibition (>95%), fibroblast viability (>85%), cytokine suppression (TNF-α ↓ 70%, IL-6 ↓ 65%), ROS reduction (≈60%)	[119,125,126]
Proliferation	Fibroblast migration, angiogenesis, collagen deposition, epithelialization	Poor moisture retention, secondary trauma	Accelerated wound closure (~80% by day 7 vs. 45% control), increased collagen deposition (1.8-fold), enhanced fibroblast migration (2-fold)	[118,127]
Maturation/Remodeling	ECM organization, collagen crosslinking, scar remodeling	Incomplete collagen alignment, excessive contraction	Sustained drug release (>14 days), reduced scar index (~40%), improved tensile strength (~30%)	[124,128]

↓ indicates a reduction relative to the control.

**Table 4:** Comparative characteristics and therapeutic efficiencies of natural polymer-based wound healing platforms.

Polymer	Distinct Characteristics	Advantages	Limitations	Typical Wound Environment/Application	Quantitative Healing Efficiency	Ref.
Chitosan	Cationic polysaccharide (β-1,4-linked D-glucosamine); electrostatic interaction with cells	Hemostatic activity, antibacterial effect, macrophage activation, collagen alignment	pH-dependent solubility, variability in deacetylation degree, limited neutral stability	Acute bleeding wounds, infected sites	Wound closure 88% (day 10), bacterial inhibition 95%, collagen deposition 1.7× vs. control	[171,172]
Gelatin	Denatured collagen with RGD motifs; peptide-based adhesion	Cell adhesion, fibroblast proliferation, angiogenesis promotion	Rapid enzymatic degradation, weak mechanical strength	Deep tissue defects, graft or granulating wounds	Wound closure 90% (day 12), fibroblast proliferation 1.6×, angiogenic index 1.5×	[96,173,174]
Alginate	Anionic polysaccharide (M/G blocks); Ca^2+^ crosslinking, ion-exchange gelation	Moisture retention, exudate absorption, autolytic debridement	Fragility from Na^+^–Ca^2+^ exchange, over-gelation risk, low elasticity, limited elasticity	Chronic exudative ulcers, moist environments	Moisture retention 85%, wound closure 90% (day 10), fibroblast density 1.4×	[96,125,172]
Fucoidan (composite use)	Sulfated polysaccharide from brown algae; negative charge with angiogenic potential	Antibacterial, antioxidant, VEGF upregulation	Ionic instability, viscosity variation, extraction cost	Ischemic or diabetic wounds	VEGF expression 2.3×, wound closure 93% (day 10), ROS reduction 40%	[175,176]
Chitosan–Alginate–Fucoidan Composite	Hybrid ionic network (CS:Alg:Fu = 2:1:0.5); calcium-crosslinked porous structure	Synergistic antibacterial and angiogenic response, high porosity and compressibility	Batch variability, Ca^2+^ exchange instability	Full-thickness, delayed-healing wounds	Collagen I/III ratio 1.9×, microvessel density 1.8×, wound closure 95% (day 10)	[177,178]
Chitosan–Gelatin Blend	Cationic–neutral polymeric matrix	Improved elasticity, moisture balance, adhesion	Thermal instability, degradation rate mismatch	Burn wounds, surgical sites	Wound closure 91% (day 12), fibroblast migration 1.7×, tensile strength 0.35 MPa	[179,180]
Alginate–Pectin–Collagen Composite	Triple-polymer hydrogel; collagen-enriched ECM mimic	ECM remodeling, anti-inflammatory activity	Limited mechanical resistance	Chronic diabetic ulcers	Wound closure 92% (day 14), collagen density 1.8×, TNF-α reduction 40%	[181,182]

## Data Availability

No new data were created for this study. All data analyzed in this review are available in the publications cited in the references section.

## Abbreviations

The following abbreviations are used in this manuscript:

PNPs	Polymer nanoparticles
RESS	rapid expansion of supercritical solutions
RESOLV	rapid expansion of supercritical solution into liquid solvent
RAFT	reversible addition fragmentation chain transfer
ATRP	atom transfer radical polymerization
PLGA	Polylactide-co-glycolide
PEG	Polyethylene Glycol
PLA	Polylactic acid
PCL	Polycaprolactone
EA	Ethyl Acetate
DCM	Dichloromethane
PVA	Poly(vinyl alcohol)
NAs	Nucleic Acids
AC	Acetonitrile
EE	Encapsulation Efficiency
W/O/W	Oil-in-Water
PEO	Polyethtlene oxide
LNPs	Nanostructured Lipid Carriers
SLNs	Solid Lipid Nanoparticle
ROS	Reactive Oxygen Species
TFH	Thin Film Hydration
EI	Ethanol Injection
REV	Reverse-Phase Evaporation Vesicle
DR	Dehydration–Rehydration
HPH	High-Pressure Homogenization
MLV	Multilamellar Vesicle
LUV	Large Unilamellar Vesicle
SCF	Supercritical Fluid
PCS	Photon Correlation Spectroscopy
US	Ultrasound
DLS	Dynamic Light Scattering
XRD	X-ray Diffraction
PLHN	Polymer–Lipid Hybrid Platforms
LbL	Layer-by-Layer assembly
NLCs	Nanostructured lipid carriers
RNA	Ribonucleic Acid
